# “An Experiment” Examined

**Published:** 1863-05

**Authors:** B. Wood


### "AN EXPERIMENT" EXAMINED.

BY DR. B. WOOD.

A recent issue of the "American Dental Review," dated for January, details "An Experiment with Wood's Metallic Filling," going to show that this material requires a temperature between 200 and 210° degrees Fahr. to render it plastic (or some fifty degrees more than is really the case) and thereupon condemning it for the purpose of filling teeth. Conceiving the points wherein the "experiment" was at fault to be sufficiently manifest, and as the simplest tests, intelligently conducted, would have sufficed to demonstrate the error educed by it, I did not think it likely to mislead, until finding it promptly copied, doubtless without examination, in the "Dental Cosmos" of the same date. But if calculated to mislead journalists, it would seem due to readers to make such a full and clear exposition as shall set the matter right. This would have been offered through the original medium, but that the "Review" is but a small phamplet or circular, irregularly issued by the parties interested, who only promise that "subsequent numbers will appear just when there appears to us to be enough matter of sufficient interest to our friends to justify the demand on their time and our pocket;" so that there is no certainty that another ever will appear.

Having melted the metal into the cavity of a tooth, the experimenter proceeds to say:

"Finding the tooth became warmer than it was thought a tooth in the mouth, with life in it, would bear, a small piece of the filling was placed on a watch-glass; this was placed on water and heat applied. At 200 degrees Fahrenheit, (according to the thermometer used,) it was solid. A few degrees above this it became plastic. At 210 degrees it was fluid." It is not expressly stated that the bulb of the thermometer was immersed in the water, but this is presumed of course, being the only means of ascertaining its temperature.

All experimentation should recognize elementary principles. To neglect these but plunges us at every step into errors under the delusion that we are demonstrating facts. The case under consideration is valuable as affording an apt illustration of this.

In heating water the lower strata become hot first, and gradually conveys the heat upwards, so that until the fluid boils, when the temperature of the mass becomes nearly uniform, the thermometer indicates a difference according to the depth at which the bulb is submerged. The difference may amount to several degrees in the space of half an inch, and if the depth be considerable the surface may be cold while the bottom is quite warm. Accordingly, the substance under examination should be immersed to the *same depth with* the blub of the thermometer. When not only at less depth but actually *on the surface* of the water, exposed to cold currents of atmosphere, the indications afforded by the heat of the fluid will be, of course, much wider from the mark. For example, the blub of the thermometer having been immersed half an inch in hot water, upon being partly withdrawn so that only the lower portion dipped into it, resulted in a falling of some fifteen degrees, and *vice versa*. (See remarks on "Determining the melting point of metals." *Journal Fr. Institute* for Jan., 1862, and "Dental Cosmos" for Feb., 1862.)

But in the "Experiment" under notice, the metal is not only exposed to the air at the surface of the fluid, the heat of which is made the criterion, but "*placed on a watch glass*" and "*this was placed on water*," thus interposing a non-conductor, cutting off the heat from below, while exposed to the cold above! That the metal so situated melted or softened at all when the water indicated 200° or 210° is pretty conclusive that the sample *was* "genuine." It is added, indeed, that the plugged tooth was inserted in the water, and the state of the filling in it coincided with the portion on the watch glass. But this seeming corroboration, (which, without attributing it to vague inference, may be accounted for by the supposition that the tooth was merely "inserted" in the water without remaining long enough to acquire its temperature, or that the water had a few moments to cool,) implies a clear contradiction, for if the water was only hot enough to soften the metal immersed in it, the metal could not have softened in a dish placed on the surface; and so of the converse. It is simply impossible that the two pieces so differently situated should coincide in temperature.

I may say, before noticing other points, that the heat at which the Plastic Metallic Filling softens is two or three degrees higher than that at which the non-plastic form of the alloy melts, and which is given by Professor Silliman at "about 158° Fahr.," but by Lipowitz, the European chemist, who, having made a series of experiments with it, (as published in the *London Chemical News*,) I was disposed to think might have been in possession of more accurate modes of determining at 140°, as chronicled in the Journals. My own measurements, according to the best methods and precautions that I have been able to devise, indicate 150° as the melting point, and the same as the point of congelation, the blub of the thermometer being immersed in the melted metal, and this allowed to cool gradually. Examined in hot water it varied, being always perfectly fluid at 160°, and always solid at 150°, but generally indicated a higher melting point by a few degrees than by the other method.

The plastic form of the metal, when examined according to the first method, remained soft down to 152°, below which it solidified around the blub. Above 160° it was clearly too fluid for moulding or packing satisfactorily into dental cavities. Examined in hot water it remained soft and plastic, sometimes at 154°, sometimes near 156°, but always below 160°, the heat of the water being kept so nearly uniform that the thermometer at no time during the operation rose above 164°

or fell below 154°. It remained soft at a few degrees lower heat than that at which it melted in the first place. All these points are open to properly conducted tests. The condition of the metal should be examined by means of a small wooden point, as it becomes plastic and in working order before it *melts down*, when it is too soft for manipulating well.

The "Review" further says: "It was not possible to keep the finger in the water at the heat requisite to the plasticity of the filling, or hold in the fingers the point of the instrument when hot enough to make the alloy plastic." This suggests a few points of interest.

In respect to endurance of heat, the finger is probably the most sensitive part of the body, and doubtless more than the mouth. It can not be borne long in water at 140°, while we can hold water in the mouth at 140°, and sip it at 150°. We drink coffee and tea at a temperature between 130° and 140°. At 130° they are rather luke-warm and insipid; at 140°, quite hot enough and more palatable. But persons accustomed to very hot drinks relish them better at a higher temperature. A bar of iron heated in water to 180°, can be held between the teeth without inconvenience, and at 208° with little discomfort. As many degrees *below* the normal standard would certainly not be less uncomfortable. It would seem as though nature had expressly accommodated the organs of the mouth to thermal variations reaching to points nearly equidistant upon either side of the blood-heat mark, (98°). Water is taken with impunity at 32° Fahr., being in certain seasons and latitudes obtainable (of nature) in the liquid state only at that temperature, which is 66° below that of the oral cavity—the corresponding limit on the other side of the scale would be indicated by 164°. These would seem to designate the boundaries between which the variations of heat may safely pendulate. The teeth will bear greater extremes without harm, often without discomfort; but, on the other hand, they are sometimes sensitive to less. Irritable dentine is usually as susceptible to heat raised to 130° as to 160°, and not less so to a corresponding reduction below the normal standard.

The "Review" surely ought not to need telling that the temperature of an instrument requisite to melt a substance, is not the same as that of the substance after being melted by it. It must have surplus heat in order to effect this. If, for example, (being equal in other respects,) the temperature of the plugger be 160°, and that of the metal 60°, making a difference of 100°, then, when brought together, equilibrium is restored, the one loses and the other gains 50°, resulting in a mean temperature of 110°. The experimenter might not like to thurst his hand in hot water, but, after it had melted its weight of ice, would not feel alarm. We can not "*hold* in the fingers the point of a plugger when hot enough to make the alloy plastic," but we can rest the finger a moment upon it so as to test its temperature, a test which, by a little practice, becomes exceedingly accurate.

The experimenter thinks because the tooth under experiment became "warm" the Filling would not answer for a "living tooth." It does not appear that the warmth harmed his "living" finger, which member is more susceptible to thermal impression than the tooth, gum or socket. The tooth-bone is a very slow conductor, so that by the time what heat it receives from the plug in it can be diffused throughout its substance, it becomes gradually accustomed to the transition, and the slight elevation of temperature in ordinary cases is not even perceptible. But if the tooth were a mere shell, it might be uncomfortable, but even in such cases I have recently employed the Filling without complaint on this score. But did any one plugging a tooth out of the mouth with foil as annealed over the spirit lamp, or with Hill's Stopping, become terrified as to the consequences because it became warm? Gold foil is "annealed" at a *red heat*, or 1000 degrees Fahr., and by the time conveyed from the flame to the tooth is thought to be cooled down enough for introduction. There is no criterion of the heat it still retains; when it hisses on touching a moist spot, it indicates the boiling point, at least, or 212° Fahr., which some recommend as a means of dissipating any moisture present in the cavity. Amalgam also is now-a-days being worked by means of a hot iron that would soften the "Metallic Filling." No one objects to Hill's Stopping on this score, yet it requires as high a heat to work it as does the metal, and is very likely to be introduced at a higher. If the impression on a sensitive tooth be more perceptible, it is owing simply to the metal's being the better conductor of the two. As to the latter, the heat can not be high enough to do harm, without the metal's becoming too fluid to work well, and by this it is within control, which is not the case with the other, since it preserves nearly the same consistency up to a much higher temperature.

It was thought worth while to notice thus closely the points raised, (which involves the sum total of objections urged to this material,) as serving to illustrate certain principles and their proper application, as well as to settle some questions of practical interest.

February 17, 1863.

---

Messrs. Editors of the Dental Register:—It is proper to add in this connection that the foregoing was written for the *Vulcanite*, but as the appearance of that journal has been delayed beyond the expected time, the editor has kindly permitted me to take a copy of it for simultaneous publication in the *Register*. This will explain the repetition of some facts already adverted to in my papers on "Metals and alloys" in this journal.

At the solicitations of relatives and friends, I shall remove the first of May to Albany, my native city, to which place communications should be addressed after this date, where they will, moreover, not be liable to miscarriage from neglect to give the precise address, as at New York, where there are so many of the same name—several of the same initial—as to occasion confusion. This circumstance alone weighed strongly in favor of selecting some neighboring point where letters would reach me if addressed simply, "B. Wood," "Dr. Wood," etc., as well as if the proper address and number of street were given—and such a point is Albany. Hereafter, therefore, I shall date from

ALBANY, New York.

Yours, &c., B. WOOD.

